# Latitude-dependent finescale turbulent shear generations in the Pacific tropical-extratropical upper ocean

**DOI:** 10.1038/s41467-018-06260-8

**Published:** 2018-10-05

**Authors:** Zhiwei Zhang, Bo Qiu, Jiwei Tian, Wei Zhao, Xiaodong Huang

**Affiliations:** 1 Physical Oceanography Laboratory/CIMST, Ocean University of China and Qingdao National Laboratory for Marine Science and Technology, 238 Songling Road, 266100 Qingdao, Shandong China; 2 Department of Oceanography, University of Hawaii at Manoa, 1000 Pope Road, Honolulu, HI 96822 USA

## Abstract

Turbulent mixing, which is critically important for the equilibrium of ocean circulation, is controlled by finescale turbulent shear (*S*^2^) of oceanic flows through shear instability. Although the relationship between *S*^2^ and mixing is well understood, the latitude-dependent generation processes of *S*^2^ remain poorly known due to the lack of geographically extensive, long-term finescale velocity measurements. Here, using one-year ADCP data from 17 moorings along 143°E, we first show that the upper-ocean *S*^2^ and its resultant mixing rate have a W-shaped latitudinal distribution in the tropical-extratropical northwest Pacific with peaks at 0–2°N, 12–14°N, and 20–22°N, respectively. Further analyses reveal that these *S*^2^ peaks are caused by vertically-sheared equatorial currents, parametric subharmonic instability of diurnal tide, and anticyclonic eddy’s inertial chimney effect, respectively. As climate model simulations are sensitive to the mixing parameterizations, our findings highlight the need to incorporate the latitude-dependent generation mechanisms of *S*^2^ to improve climate models’ prediction capabilities.

## Abstract (web summary)

Turbulent mixing is critically important for ocean circulation, but their latitudinal distribution are poorly known. Here, the authors show that the generation mechanism of the W-shaped distribution of turbulent mixing in the tropical-extratropical Pacific is latitude dependent.

## Introduction

Turbulent mixing across density surfaces (i.e., diapycnal mixing) plays a fundamental role in redistributing momentum, heat, nutrients, carbon and other materials in the ocean that modulate the ocean circulation, the biogeochemical cycles, and the long-term climate^1,2^. Because the turbulent processes are too small (with vertical scale of 10^−2^–10^1^ m) to be resolved by climate models, their mixing effects must be properly represented, or parameterized, in terms of model’s resolved features. Existing model studies have demonstrated that a wide range of the simulated climate phenomena are sensitive to the geography of turbulent mixing and its parameterizations^3–6^. A better understanding of the turbulent mixing processes, therefore, is a prerequisite for improving model’s parameterizations and its capability to accurately simulate the present and future climate.

Because of the diverse dynamical regimes, the governing physics of turbulent mixing can have a strong latitude dependence in the world ocean^7–9^. While critical for improving the model’s parameterization schemes, quantitatively understanding this latitude dependence has long been a challenging problem due to the sparsity of in situ observations^2,10^. Although ship-based dyed tracer tracking^11^ and microstructure profiling (at vertical scale of centimeters)^7^ are two direct ways to accurately measure the turbulent mixing, high-cost and sophisticated operations have limited their spatiotemporal coverage in the ocean^12^. Given the extremely patchy and intermittent nature of turbulent mixing, such direct measurements are far from adequate to quantify its latitude-dependent generation mechanisms. Alternatively, the strength of turbulent mixing can be inferred from finescale vertical shear of velocity at vertical scale of 10–100 m (termed finescale turbulent shear hereafter), that relies either on the internal wave-wave interaction theory^13,14^ or on the Richardson number-based criteria of shear instability^15,16^. Observational and theoretical studies have suggested that the turbulent dissipation rate scales with the finescale turbulent shear, while the rate of turbulent mixing (i.e., diapycnal diffusivity) is related to the former through a nearly constant mixing efficiency^17,18^. In contrast to the direct mixing measurements, finescale turbulent shear data are much easier to obtain owing to the widespread use of Acoustic Doppler Current Profiler (ADCP) measurements. For example, lowered ADCP shear data have been successfully used to infer the diapycnal diffusivity (*K*_ρ_) in different parts of the world ocean^14,19^. Based on finescale shear and strain data from thirty thousand hydrographic profiles, the most recent study of Kunze et al.^20^ proffered a rough geography of *K*_ρ_ with a nearly global scope. However, these results are based mostly on snapshots and lack of the long-term time series has limited their representativeness and the physical knowledge behind them.

Compared with the instantaneous lowered and shipboard ADCP measurements, moored ADCPs are more effective to continuously observe the oceanic finescale velocity profiles at fixed locations. Because the moored ADCP can repeatedly collect high-frequency (with sampling intervals at tens of minutes) velocity profiles for a long period (several months or longer), it allows to precisely distinguish motions with different frequencies and hence to investigate the underlying sources generating the finescale turbulent shear and its resultant mixing^21–23^. Based on moored ADCP data, many studies have reached a consensus that near-inertial waves (NIWs) and internal tides (ITs) are two primary sources for turbulent shear generation in the ocean interior^21,24–26^. In addition, some studies found that turbulent shear may also be produced by the strongly sheared mean current, such as the South Equatorial Current (SEC) and Equatorial Undercurrent (EUC)^27^, generated by Tropical Instability Waves near the equator^16,28^, and modulated by mesoscale eddies through eddy-wave interactions^29,30^. However, nearly all the previous moored ADCP-based studies deployed only one or several moorings in a limited area; as a result, it remains largely unclear how the turbulent shear/mixing and its governing physics vary with the latitude-dependent dynamical regimes in the ocean.

The tropical-extratropical northwest Pacific Ocean is featured by three distinct dynamical regimes from 0° to ~25°N^31^: the equatorial regime (0–6°N) with alternating zonal jets and strong eddy kinetic energy (EKE), the off-equatorial regime (7–18°N) with a broad and stable westward-flowing current and weak EKE, and the subtropical regime (19–25°N) with weak mean current but enhanced EKE. To investigate the characteristics of, and interactions among, the multiscale dynamical processes in these distinct regimes, the Northwestern Pacific Eddies, Internal waves and Mixing Experiment (NPEIM) was initiated between 2015–2016. As the key component of NPEIM, a mooring array consisting a total of 17 ADCP moorings were deployed along the 143°E meridian for over one year (see Methods). Extending from 0° to 22°N, the mooring section traverses the above three dynamical regimes and provides us with a unique opportunity to investigate the latitudinally dependent processes underlying the finescale turbulent shears.

Based on the year-long ADCP measurements from the NPEIM, this study for the first time reveals that in the tropical-extratropical northwest Pacific upper ocean (100–400 m layer), the finescale turbulent shear and the parameterized mixing rate are elevated at the latitude bands of 0–2°N, 12–14°N, and 20–22°N. We further find that mechanisms of the three mixing peaks are associated with strong sub-inertial shear of the equatorial currents, Parametric Subharmonic Instability (PSI) of diurnal ITs, and anticyclonic eddy’s chimney effect on wind-driven NIWs, respectively.

## Results

### Moored observations

**Fig. 1:**
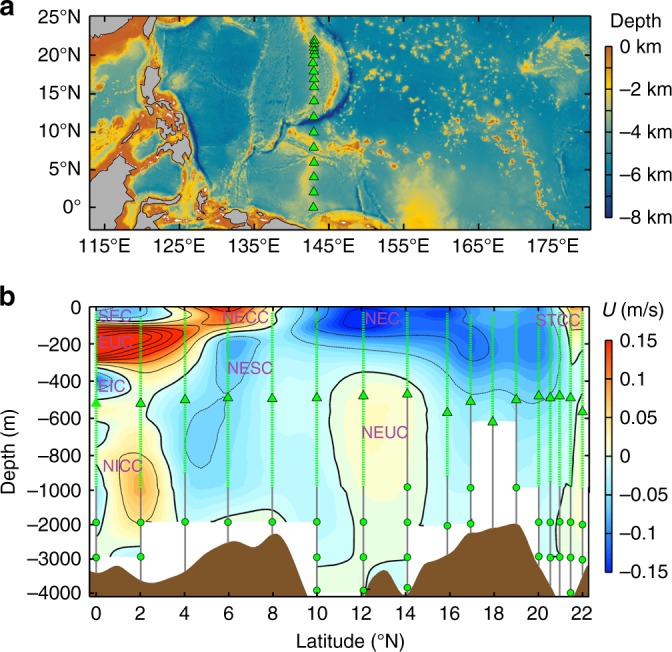
Observation sites and mean circulation. **a** Locations of the NPEIM mooring array (green triangles). Color shading shows the ETOPO1 bathymetry in the northwestern Pacific (data from https://www.ngdc.noaa.gov/mgg/global/). The maps in this figure are generated by MATLAB R2013a with M_Map (a mapping package, http://www.eos.ubc.ca/~rich/map.html). **b** Latitude-depth plot of the mooring-observed mean zonal velocity along 143°E (colors and black contours). Black thick solid, thin solid and thin dashed contours denote the zero, positive and negative velocities, respectively, with the contour interval of 0.05 m/s. Green triangles and dots indicate positions of ADCPs and current meters on each mooring, respectively, and green dashed lines indicate the observation range of ADCPs. Brown shading indicates the bottom topography along 143°E based on the half-degree smoothed ETOPO1 data. Notice that vertical scale between 1000–4000 m is different from that in the upper 1000 m. Names of the zonal currents are marked in the figure (purple words). The acronyms SEC, EUC, EIC, NICC, NECC, NESC, NEC, NEUC, and STCC represent South Equatorial Current, Equatorial Undercurrent, Equatorial Intermediate Current, North Intermediate Countercurrent, North Equatorial Countercurrent, North Equatorial Subsurface Current, North Equatorial Current, North Equatorial Undercurrent, and Subtropical Countercurrent, respectively

As a component of NPEIM to investigate the characteristics of the multiscale processes and their interactions in the northwestern Pacific, a total of 17 bottom-anchored moorings were deployed roughly along the 143°E meridian from 0° to 22°N (Fig. 1). The 17 moorings were deployed between 24/Nov/2015–04/Jan/2016 and successfully recovered between 10/Feb–08/Mar/2017, providing us with more than one-year (13–15 months) continuous moored data. Spatially, the moorings are set up at every 2° between 0–16°N (inclusive), every 1° between 16–20°N, and every 0.5° between 20–22°N, respectively. The enhanced mooring resolution between 20–22°N is to resolve the mesoscale eddies in the unstable Subtropical Countercurrent (STCC) region. Prior to the NPEIM experiment, mooring P17 at the northern end of the mooring array (~22°N) was deployed for 17 months, and totally, P17 provided continuous measurements for more than 32 months (~2.7 years). All the moorings were equipped with ADCPs, recording current meters (RCMs), and temperature chains to measure current velocity and temperature over nearly the whole water column (see Methods and Supplementary Table 1). Specifically, an upward-looking and a downward-looking 75 kHz ADCPs (only one upward-looking ADCP at P8 and P10-12) were mounted at ~500 m depth of the mooring to observe the high-frequency (half-hourly) current velocity in the upper 1000 m (or 500 m) with a vertical bin of 16 m. The high-frequency ADCP data are used to analyze the characteristics and physics of the finescale turbulent shear in this study.

As shown in Fig. 1b, the moored observations have captured the three dynamical regimes with diverse characteristics of circulation and EKE (Supplementary Fig. 1). In the equatorial regime (0–6°N), the circulation is characterized by multiple strong zonal jets, including the westward-flowing SEC and eastward-flowing North Equatorial Countercurrent (NECC) near the surface, the eastward-flowing EUC in the subsurface, and the westward-flowing Equatorial Intermediate Current (EIC) in the intermediate layer, etc. These zonal currents exhibit large velocity shears in both vertical and meridional directions and strong mesoscale EKE throughout the upper 1000 m layer. The off-equatorial regime (7–18°N), which straddles the boundary between the tropical and subtropical wind-driven gyres, is occupied by the broad and deep-reaching westward-flowing North Equatorial Current (NEC). Due to the lack of sign change in gradient of potential vorticity, the NEC in this band is dynamically stable, showing rather weak EKE above 300 m. It is noteworthy that beneath the NEC (below 400 m) between 12–14°N, there is a relatively weak eastward-flowing zonal jet. This subthermocline zonal jet was recently named the North Equatorial Undercurrent and was proposed to be generated by eddy-eddy interactions^32–34^. The northern part of the mooring array (19–22°N) is located within the southern half of the wind-driven subtropical gyre. Within this subtropical regime, there exists the relatively weak and shallow eastward-flowing STCC. Because of the baroclinic instability between the vertically sheared STCC and NEC^35^, this band displays strong EKE in the upper 300 m layer.

### Latitudinal variation

**Fig. 2:**
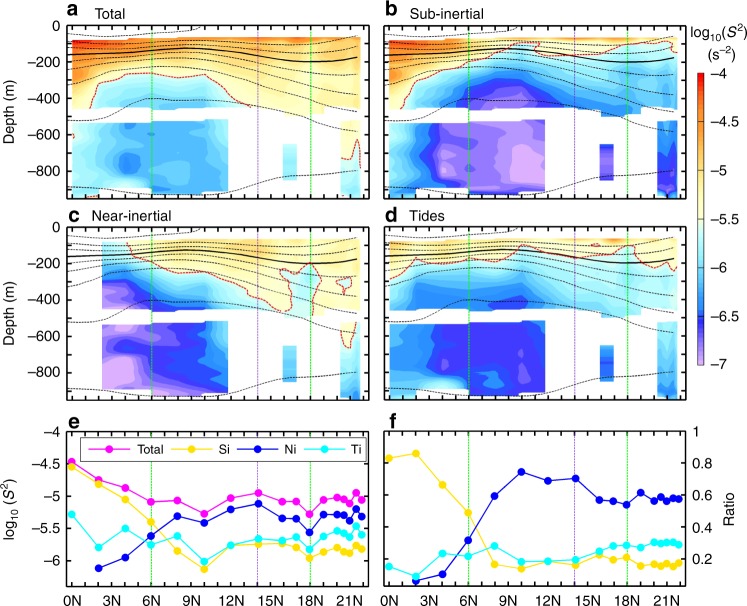
Spatial distribution of finescale turbulent shear. Latitude-depth section of finescale turbulent shear squared (*S*^2^) for **a** the total and **b**–**d** the sub-inertial, near-inertial, and tidal component, respectively. Red dashed line denotes the *S*^2^ contour of 10^−5.5^ s^−2^. Black contours denote the mean isotherms every 3 °C, among which the thick solid one is the 20 °C isotherm. Green dashed lines delineate the three different dynamical regimes and the purple dashed line marks the 14°N latitude. **e** Latitudinal distribution of the upper layer-averaged *S*^2^ (between 100–400 m) in different frequency bands. The total *S*^2^, and the sub-inertial, near-inertial, and tidal component are denoted by magenta, yellow, blue, and cyan dotted lines, respectively. **f** Same as **e** but for the ratio of each *S*^2^ component to the total one

The total finescale turbulent shear squared (*S*^2^) calculated based on the ADCP data (see Methods) displays strong heterogeneities along the mooring section (Fig. 2a). The most turbulent region occurs in the upper 400 m layer of the equator, where the largest mean *S*^2^ exceeds 10^−4^ s^−2^ near the surface. With the increasing latitude, the upper-layer (hereafter, it means the 100–400 m layer) *S*^2^ sharply decreases from the equatorial to the off-equatorial regime and then shows moderate variations further north in the subtropical regime. Vertically, *S*^2^ generally decreases with depth but the large-valued *S*^2^ can extend deeper in the subtropical regime than the two tropical regimes. For example, large *S*^2^ exceeding 10^−5.5^ s^−2^ is only confined to the upper 300 m between 4–10°N, while it can penetrate below 700 m between 20–22°N. From the 100–400 m layer-averaged *S*^2^ (excluding the mixed layer above 100 m), it can be clearly seen that the upper-layer *S*^2^ has a distinct W-shaped distribution from equator to 22°N. Three peaks of the “W” are located at 0–2°N, 12–14°N, and 20–22°N, respectively, and they fall into the three dynamical regimes separately.

To obtain the physical insights into the latitudinal variation of *S*^2^, we decompose the finescale turbulent shear into three frequency-dependent components, that is, sub-inertial, near-inertial, and tidal components, respectively (Fig. 2b–e; see Methods). Generally, the sub-inertial *S*^2^ shows a distribution similar to the total *S*^2^ in the equatorial regime, whereas this correspondence applies to the near-inertial *S*^2^ in the off-equatorial and subtropical regimes. The upper layer-averaged sub-inertial *S*^2^ is quite close to the total one in the equatorial regime, with its contribution exceeding 83% between 0–2°N (Fig. 2f). However, the strong sub-inertial *S*^2^ only occurs near the equator and it rapidly weakens with increasing latitude. The ratio of the sub-inertial *S*^2^ over the total *S*^2^ is generally lower than 20% in the off-equatorial and subtropical regimes. In contrast to the sub-inertial component, the near-inertial *S*^2^ is rather weak in the equatorial regime, but keeps increasing until it reaches its first peak at 14°N. North of 14°N, the near-inertial *S*^2^ first decreases to a local minimum at 18°N and then increases to its second peak at 21.5°N. In the off-equatorial and subtropical regimes, the near-inertial *S*^2^ accounts for 54–74% of the total *S*^2^, with the highest and lowest percentage at 10°N and 18°N, respectively. Compared with the sub-inertial and near-inertial components, the upper-layer tidal *S*^2^ is relative weak with a low ratio (<30%) throughout the section. This observed result is consistent with the existing knowledge that in the ocean interior away from rough topography, the ITs are generally dominated by low vertical modes that have weak velocity shears^2^. From the results in Fig. 2, we can conclude that the upper-layer *S*^2^ is dominated by the sub-inertial component in the equatorial regime, and the near-inertial component in the off-equatorial and subtropical regimes, respectively. In the following, we explore the generation mechanisms of the finescale turbulent shear in each of the dynamical regimes.

### Equatorial regime

**Fig. 3:**
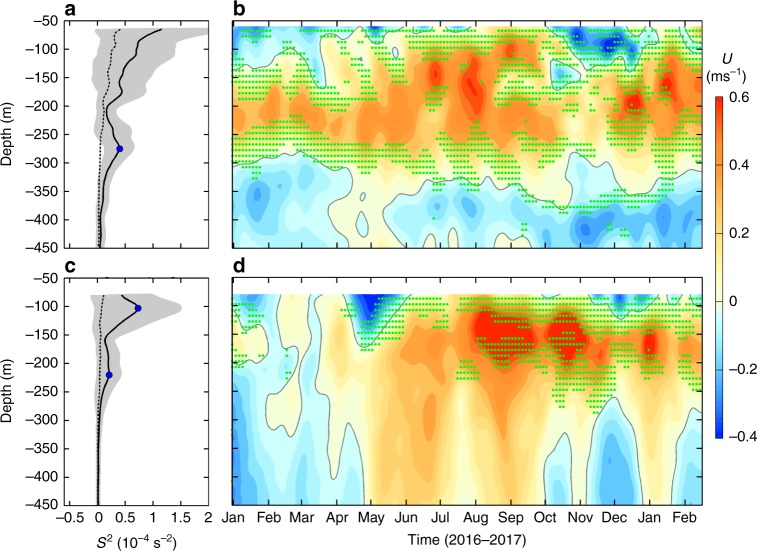
Sub-inertial *S*^*2*^ and zonal velocity at 0–2°N. **a** Mean vertical profile of the sub-inertial *S*^2^ at 0° (black solid line). Gray shading denotes standard deviation of the mean profile and blue dot marks position of the local maxima. Black dashed line denotes the mean *S*^2^ associated with the 20–100 day equatorial waves. **b** Time-depth plot of the sub-inertial zonal velocity at 0°. Gray line denotes the zero contour of velocity. Green dots indicate the positions where shear squared of the sub-inertial zonal velocity exceeds 10^−4.8^ s^−2^. **c**, **d** are the same as **a**, **b**, respectively, but for the 2°N

In the strong finescale shear region at 0° (2°N), the mean sub-inertial *S*^2^ has two local maxima in the upper layer, one near the surface (at ~100 m) and the other at ~270 m (220 m) depth (Fig. 3a, c). The vertical positions of these two maxima correspond well with the interface of SEC/EUC and EUC/EIC, respectively (Fig. 3b, d), demonstrating that the observed large sub-inertial *S*^2^ in the equatorial regime is closely associated with the vertically sheared zonal currents. In addition to the mean current, the equatorial waves such as Yanai waves and internal gravity waves can also make important contributions to the sub-inertial *S*^2^ near the equator^36,37^. Indeed, strong 20–100 day modulations can be clearly seen from the time series of sub-inertial shear (Supplementary Fig. 2a). Averaged over the observation period, the upper-layer ratio between the 20–100 day and total sub-inertial *S*^2^ is 0.34 (0.31) at 0° (2°N), suggesting that equatorial waves roughly account for one third of the sub-inertial *S*^2^ near the equator (Fig. 3a, c).

**Fig. 4:**
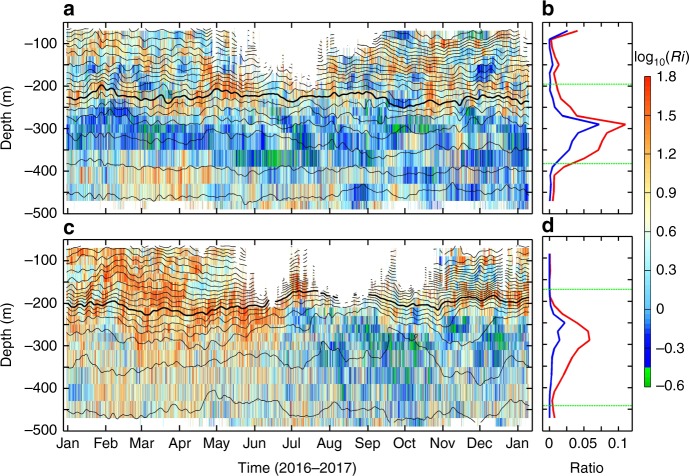
Richardson numbers in the equatorial regime. **a** Time-depth plot of Richardson number (*Ri*, colors) and temperature (black contours) at 0°N. The potential shear instability events with *Ri* < 1/3 are marked with green colors. Contour interval of temperature is 1 °C and the thick line denotes the 15 °C isotherm. **b** Depth-dependent occurrence ratio of *Ri* < 1/3 over the observation period (red line). The blue line is same as the red one except that the sub-inertial *S*^2^ was used when calculating *Ri*. Green dashed lines indicate the core depths of EUC and EIC. **c**, **d** are the same as **a**, **b**, respectively, but for 2°N

In order to qualitatively evaluate how the strong *S*^2^ in the equatorial regime can impact turbulent mixing, we further examine the possibility of shear instability based on Richardson number (see Methods). Here, the criterion of *Ri *< 1/3, rather than the traditional *Ri *< 1/4, was used to identify the possible occurrence of shear instability, because recent studies found that the equatorial currents are marginally unstable and turbulence can occur even with slightly higher *Ri* values^16,38^. Despite that the relatively coarse vertical resolution of the measurements (~20 m) may overestimate the *Ri*^15^, we can still see that at equator, a large number of potential instability events (with *Ri *< 1/3) intermittently occur beneath the EUC core at ~200 m (Fig. 4a). The largest occurrence frequency of potential instabilities is exactly located at the EUC/EIC interface with its value as high as 0.11 (Fig. 4b). Compared with the EUC/EIC interface, the potential instability is suppressed at the SEC/EUC interface due to the stronger stratification despite the fact that *S*^2^ there is stronger. If only the sub-inertial *S*^2^ was used when calculating the *Ri*, the frequency of potential instabilities does not decrease significantly and the largest occurrence frequency can still reach 0.07. This result implies that even without the high-frequency internal waves, the shears generated by the mean current and sub-inertial waves alone can result in strong mixing at the equator. Similar to the equator, *Ri* at 2°N is also lower beneath the EUC core than above it (Fig. 4c). Although *Ri* at 2°N is generally higher than the equator because of the decreased *S*^2^, the occurrence frequency of *Ri *< 1/3 at the EUC/EIC interface still exceeds 0.05 (Fig. 4d).

### Off-equatorial regime

**Fig. 5:**
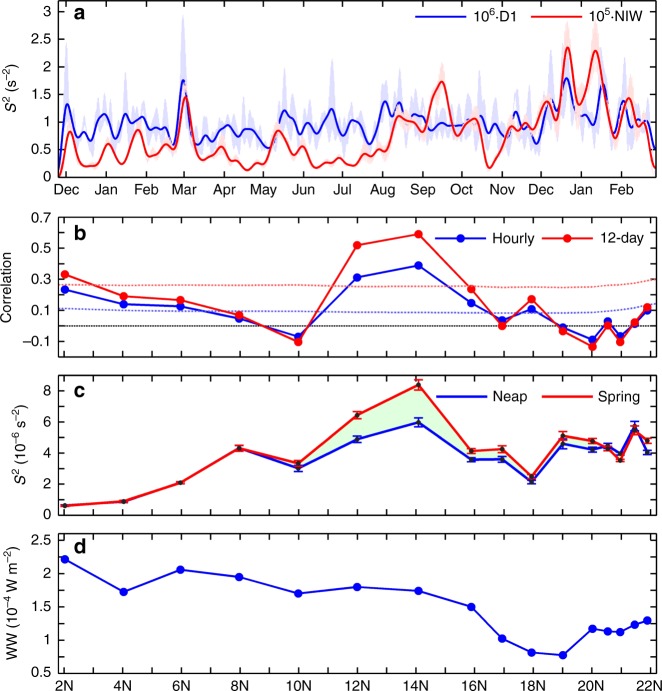
Comparisons between *S*^2^ in the near-inertial and diurnal bands and the near-inertial wind work. **a** Time plots of the upper layer-averaged near-inertial *S*^2^ (in red, multiplied by 10^5^) and diurnal *S*^2^ (in blue, multiplied by 10^6^). Dark and light colors show the 12-day low-pass filtered and unfiltered time series, respectively. **b** Latitudinal distribution of correlation coefficient between upper layer-averaged near-inertial and diurnal *S*^2^. Red and blue dotted lines are results for the 12-day low-pass filtered and unfiltered *S*^2^, respectively. Red and blue dashed lines denote the corresponding 95% significance levels from the Monte Carlo simulation. Black dashed line denotes the zero value. **c** Mean near-inertial *S*^2^ of the top half-diurnal (red) and lower half-diurnal-*S*^2^ periods (blue) during each 14-day spring-neap cycle. Error bar is the 95% confidence interval computed using the bootstrap method. **d** Mean near-inertial wind work during the observation period calculated based on the slab model (see Methods)

Compared with the equatorial regime, the strength of finescale turbulent shear is overall weaker in the broad off-equatorial regime of 7–18°N. An exception is at 12–14°N, where the upper layer-averaged *S*^2^ is 76–110% larger than the trough value (i.e., at 10°N and 18°N) in this regime (recall Fig. 2e). This turbulent shear peak is primarily attributable to the near-inertial *S*^2^, whose magnitude is strongest among all the observation sites. To examine whether this near-inertial *S*^2^ peak is caused by wind forcing, which has long been thought as a primary generation source for NIWs in the upper ocean^10^, we calculated the wind work (WW) on the near-inertial motions based on the slab mixed-layer model (see Methods). Different from the observed latitudinal distribution of near-inertial *S*^2^, the near-inertial WW only shows moderate values between 12–14°N (Fig. 5d)^39,40^. Because the long-distance propagating NIWs from far field are dominated by low modes and contain weak shears, this near-inertial peak is also unlikely caused by the NIWs originating from midlatitude storm track regions^10^. The above results indicate that there must be other mechanisms responsible for the near-inertial *S*^2^ peak at 12–14°N.

Near and equatorward of the critical latitude where the IT frequency is twice the local inertial frequency, PSI can be an effective way to generate higher-mode NIWs^41,42^. For the K_1_ and O_1_ diurnal ITs, their critical latitudes are around 13.4°N and 14.5°N, respectively, coinciding well with the 12–14°N near-inertial *S*^2^ peak. To investigate whether this *S*^2^ peak is associated with PSI, we compared the shears relating to the diurnal ITs and NIWs in Fig. 5. In connection to the near-inertial *S*^2^ peak, the diurnal and near-inertial *S*^2^ show high correlations at 14°N and 12°N (with correlation coefficients of 0.59 and 0.52, respectively; Fig. 5b), where the averaged near-inertial *S*^2^ during the high-diurnal-shear period is 30–40% larger than during the low-diurnal-shear period (Fig. 5c). However, no such significant correlations between the diurnal and near-inertial *S*^2^ can be found at the other latitudes. Given that the PSI-related NIW generation is only active near and slightly equatorward of the critical latitude, the results in Fig. 5 provide evidence for the occurrence of PSI.

**Fig. 6:**
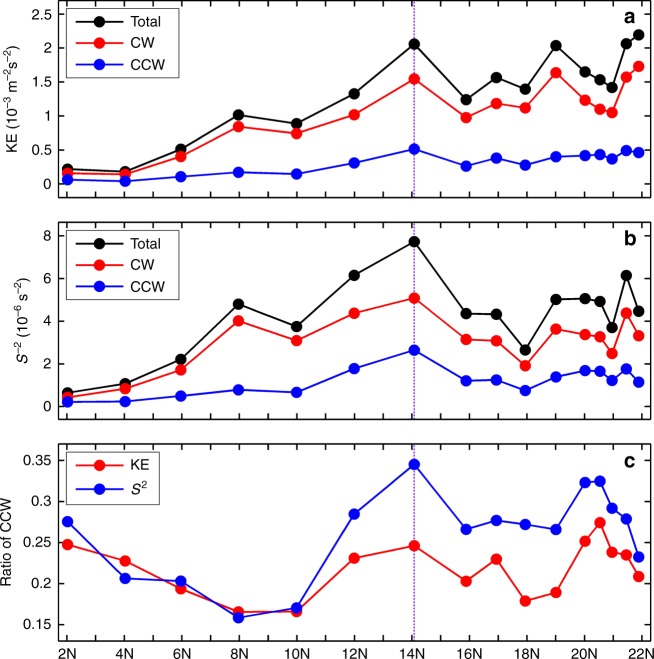
Latitudinal distribution of the mean near-inertial KE and *S*^2^. **a** Black, red and blue lines denote the total, CW and CCW near-inertial KE (averaged over 100–400 m), respectively. Purple vertical line indicates the PSI latitude at 14°N. **b** is the same as **a** but for the *S*^2^. **c** Ratio between the CCW KE (red line) and *S*^2^ (blue line) to the total ones

Different from the wind-generated NIWs, whose energy primarily propagates downward from the surface, the PSI-generated NIWs has comparable up-going and down-going energy^42^. By decomposing the near-inertial motions into clockwise (CW) and counter-clockwise (CCW) rotating components with increasing depth (whose energy goes downward and upward, respectively; see Methods), we find that both the upward-going CCW near-inertial kinetic energy (KE) and *S*^2^ have a peak at 12–14°N (Fig. 6a, b). The elevated CCW KE and *S*^2^ are more noticeable from their ratios to the total ones, lending additional evidence for PSI (Fig. 6c). It is noteworthy that the ratio of CCW *S*^2^ to the total is larger than that of KE (e.g., 0.35 vs. 0.25 at 14°N), which agrees with the knowledge that PSI-generated NIWs are dominated by higher-mode waves. If we assume that the CCW NIWs are totally from PSI and that the PSI-generated NIWs have equal KE and *S*^2^ for the CW and CCW components, the ratio in Fig. 6c would mean that PSI explains 50% (46%) of the KE and 70% (57%) of *S*^2^ in the near-inertial band at 14°N (12°N).

### Subtropical regime

Unlike the equatorial and off-equatorial regimes where NIWs are generally weak except at the PSI critical latitudes (i.e., 12–14°N), the near-inertial KE and *S*^2^ are strong in the whole subtropical regime (Fig. 2c, e; Fig. 6a, b). The enhanced NIW activities, nevertheless, do not coincide with the weak near-inertial WW here (Fig. 5d)^39,40^, indicating that other factors may concentrate the wind-generated NIWs and increase their vertical wavenumber, or that there exist additional energy sources for NIWs. Previous theoretical and modeling studies have suggested that mesoscale eddies play significant roles in modulating the NIW activities through eddy-NIW interactions^10,43–45^. Considering that the subtropical regime is abundant with mesoscale eddies (strongest among the three regimes, Supplementary Fig. 1), it is natural to ask whether the eddy-NIW interaction is responsible for the strong near-inertial KE and *S*^2^ here. The mesoscale eddy-resolving mooring array in the subtropical regime gives us a unique opportunity to answer this question (Fig. 1).

**Fig. 7:**
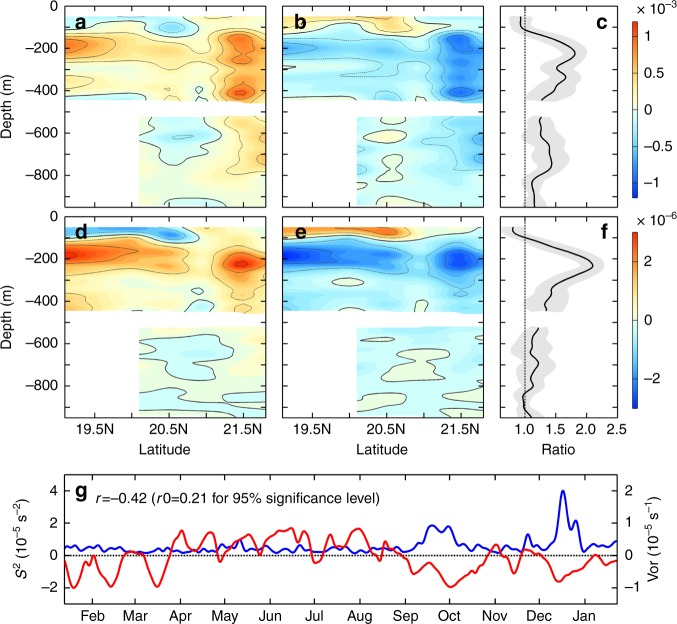
Influence of subtropical eddies on the near-inertial KE and *S*^2^. Latitude-depth section of the mean near-inertial KE (unit in m^2^ s^−2^) during **a** AE and **b** CE periods. Notice that to highlight the difference between AE and CE periods, the annual mean near-inertial KE has been removed in **a**–**b**. Gray lines are the KE contours with an interval of 2.5 × 10^−4^ m^2^ s^−2^ and the thick gray line denotes the zero contour. **c** Depth-dependent ratio between **a** and **b** averaged over the subtropical section (black line). Gray shading denotes the standard deviation of the mean ratio. Black vertical line indicates the value of 1. **d**–**f** are the same as **a**–**c**, respectively, but for the near-inertial *S*^2^ (unit in s^−2^). The gray contours have an interval of 1.0 × 10^−6^s^−2^. **g** Time series of the upper layer-averaged near-inertial *S*^2^ (blue, vertical axis on the left) and altimeter relative vorticity (red, vertical axis on the right) at mooring P16. Black horizontal line indicates the zero value. For a better view, both the *S*^2^ and vorticity have been 5-day low-pass filtered. Correlation coefficient between their time series is marked in the top left corner

In Fig. 7a–f we show the mean near-inertial KE and *S*^2^ when the moorings are influenced by anticyclonic eddies and cyclonic eddies (AEs and CEs; see Methods), respectively. It reveals that the near-inertial KE during the AE-impacted periods is significantly enhanced between 100–800 m when compared with the CE-impacted periods. The largest KE difference between AE-impacted and CE-impacted periods occurs at ~230 m where their averaged ratio is as high as 1.8 (Fig. 7c). The AE/CE ratio for KE gradually decreases with depth below 230 m, but it can still exceed 1.4 at ~700 m. With respect to the near-inertial *S*^2^, its enhancement in connection with the AEs is even clearer between 100–400 m and the AE/CE ratio reaches 2.1 at ~230 m (Fig. 7f). This modulation of near-inertial *S*^2^ by mesoscale eddies can be better seen from its strongly negative correlation with the surface relative vorticity (*ξ*) that negative *ξ* significantly elevates the near-inertial *S*^2^ (Fig. 7g). These observed phenomena are well consistent with the existing theory that AEs with negative *ξ* act as “inertial chimneys” that can trap the wind-generated NIWs by lowering the local effective inertial frequency ($f_{\mathrm{{eff}}} = f_0 + \xi /2$, *f*_0_ is local inertial frequency)^43–46^. Specifically, as the AEs (CEs) lower (raise) the *f*_eff_, they, on one hand, broaden (narrow) the waveband for NIW generations and, on the other hand, trap (exclude) the NIWs generated inside, therefore greatly increasing (decreasing) the near-inertial KE and *S*^2^ within them^43,46^. Additionally, because *ξ* of the baroclinic AEs rapidly weakens with depth and becomes quite small below 200 m (less than −0.03*f*_0_; Supplementary Fig. 3), the trapped NIWs with a frequency close to and lower than *f*_0_ approach the critical layer near 200 m where their downward group velocity diminishes^43^. Consequently, the NIWs’ energy converges and the vertical wavenumber increases near the critical layer, resulting in the observed KE and *S*^2^ peak at ~230 m within AEs. It deserves to note that in the deeper layer between 500–950 m, the AE/CE ratio for *S*^2^ is quite smaller than KE with their respective mean values of 1.1 and 1.3 (Fig. 7c, f). This result suggests that although AEs can facilitate the downward propagation of NIWs, the deep-reaching waves are primarily in low modes and have relatively weak vertical shears.

The same analysis procedure mentioned above is also performed on the 2.7-year-long ADCP data at mooring P17, which captured 7 AEs and 6 CEs during the whole observation period. The long-term observation reveals similar results as in Fig. 7, demonstrating the robustness of the statistical results, i.e., the enhanced near-inertial KE and *S*^2^ within AEs due to the inertial chimney effect (Supplementary Fig. 4a–b). We should note that this chimney effect applies only to the down-going NIWs, since no difference of the CCW-component KE and *S*^2^ are found between AEs and CEs (Supplementary Fig. 4c–d). Although AEs and CEs play opposite roles in organizing the NIWs, their net effect on near-inertial *S*^2^ is not zero but tends to elevate it due to the existence of critical layers within AEs. This nonzero net effect can be inferred from the observed result that the time-mean *S*^2^ has a more similar distribution to the mean *S*^2^ within AEs than CEs (Supplementary Fig. 4b)^47^. The inertial chimney effect of AEs therefore at least partly explains the enhanced near-inertial *S*^2^ in the subtropical upper ocean, where WW is generally weak. In addition to the AEs’ inertial chimney effect, there may also be other reasons for the strong near-inertial shear here that will be discussed in the discussion section.

### Diapycnal diffusivity

**Fig. 8:**
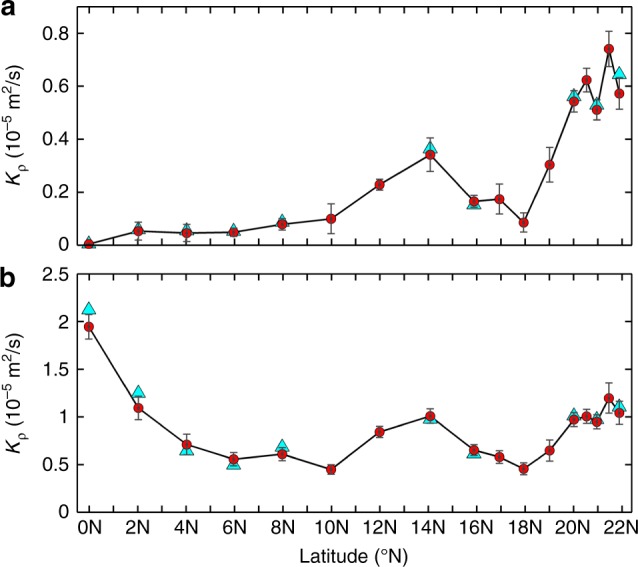
Latitudinal distribution of the finescale parameterized *K*_ρ_. **a** and **b** are the upper layer-averaged *K*_ρ_ estimated from the GHP and *Ri*-based methods, respectively. Cyan triangles and red circles denote the results based on moored and Argo stratifications, respectively. Gray error bars denote the 95% confidence interval computed using the bootstrap method

Given that the diapycnal diffusivity (*K*_ρ_) associated with finescale turbulent shear is of broad interest, it is meaningful here to estimate *K*_ρ_ and evaluate its latitudinal variation. To achieve this, we adopted two independent finescale parameterization methods based on the observed velocity shear (see Methods). The annual mean *K*_ρ_ estimated based on the Gregg–Henyey–Polzin (GHP) and *Ri*-based parameterization methods are shown in Fig. 8a, b, respectively. For *K*_ρ_ from the *Ri*-based method, it displays a similar W-shaped latitudinal distribution to *S*^2^ (Fig. 8b vs. Fig. 2e), which is not surprising given the strong dependence of *Ri* on *S*^2^. The three *K*_ρ_ peaks at 0, 14°N, and 21.5°N reach 19 × 10^−6^ m^2^ s^−1^, 10 × 10^−6^ m^2^ s^−1^, and 12 × 10^−6^ m^2^ s^−1^, respectively, and they are 4.2, 2.2, and 2.7 times the trough values at 10°N and 18°N (about 4.5 × 10^−6 ^m^2 ^s^−1^). With respect to the GHP parameterized *K*_ρ_, it generally has a similar distribution to the *Ri*-based result between 10–22°N (Fig. 8a vs. Fig. 8b). The peak value of the GHP *K*_ρ_ at 14°N (21.5°N) reaches 3.4 × 10^−6 ^m^2 ^s^−1^ (7.4 × 10^−6^ m^2^ s^−1^) and is 4.0 (8.7) times the trough value at 18°N (i.e., 0.85 × 10^−6^ m^2 ^s^−1^). In the equatorial regime, however, the GHP parameterized *K*_ρ_ is only between 0.04–0.5 × 10^−6^ m^2^ s^−1^ due to the very small latitude-correction term near the equator (i.e., $j\left( {\frac{f}{N}} \right)$ in the parameterization; see Methods). The low parameterized diffusivities in the equatorial regime are obviously unrealistic not only because they are one to two orders of magnitude smaller than the previously observed *K*_ρ_ in the similar regions^48–50^, but also because the high frequency of potential shear instabilities is found here (recall Fig. 4). These results are actually consistent with Liu et al.’s^50^ analysis that in the equatorial region the turbulent shear (also mixing) is dominantly caused by the sub-inertial currents (recall Fig. 2), and therefore the principle of the GHP parameterization based on the internal wave-wave interaction theory is violated there. Considering that the *Ri*-based estimates of *K*_ρ_ are much closer to the observed results in the equatorial regime (both on order of 10^−5^ m^2^ s^−1^), we suggest that the *Ri*-based parameterization (see Methods) that does not rely on specific turbulent shear processes may be more suitable to parameterize *K*_ρ_ in the equatorial regime. Even in the 10–22°N latitude range, the GHP-based *K*_ρ_ is also 40–80% lower than the *Ri*-based result. This discrepancy is likely attributable to the uncertainty of the presumed constant referenced diffusivity in the *Ri*-based parameterization that may vary in different regimes (see Methods). However, this does not influence our main conclusions since it is the mean pattern of *K*_ρ_, rather than its precise value, that is emphasized in this study.

## Discussion

**Fig. 9:**
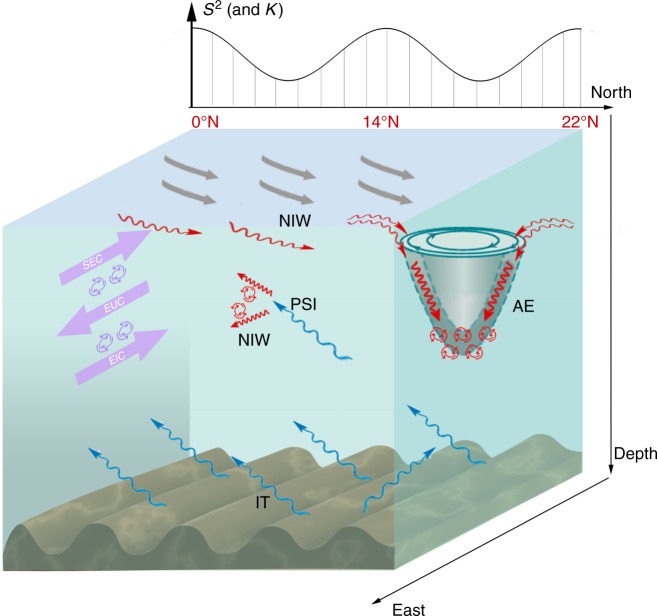
Schematic diagram of the turbulent mixing generations. Elements on the left (purple arrows), middle (marked with PSI) and right (marked with AE) of the figure represent the turbulent shear/mixing generations associated with vertically sheared equatorial currents, PSI and AE’s inertial chimney effect, respectively. The corresponding three *S*^2^ (and *K*_ρ_) peaks are marked by black curve on the top. Gray, purple, red and blue arrows denote the surface winds, equatorial currents (including SEC, EUC, and EIC), NIWs and ITs, respectively. The large swirls on the right (in green) denote the AE and the small swirls (in purple and red) denote turbulent mixing. The acronyms SEC, EUC, EIC, NIW, PSI, IT, and AE represent South Equatorial Current, Equatorial Undercurrent, Equatorial Intermediate Current, near-inertial wave, parametric subharmonic instability, internal tide, and anticyclonic eddy, respectively

By analyzing the year-long simultaneous ADCP velocity data from 17 moorings in the NPEIM experiment (between 0–22°N, along 143°E), we detect that the finescale turbulent shear and its parameterized *K*_ρ_ in the tropical-extratropical northwest Pacific upper ocean have a W-shaped latitudinal distribution with three peaks at 0–2°N, 12–14°N, and 20–22°N, respectively. The respective mechanisms accounting for the three *S*^2^ and *K*_ρ_ peaks are schematically summarized in Fig. 9 and are further discussed below.

In the equatorial regime, the strong *S*^*2*^ peak (0–2°N) is dominated by the sub-inertial shears of the equatorial zonal jets, including the SEC, EUC, and EIC. For the SEC/EUC interface, it has long been recognized to be a hotspot of turbulent shear and mixing by previous studies^51,52^. However, this study finds that the EUC/EIC interface also displays enhanced *S*^2^ due to the reversing currents. Because of the reduced stratification, *Ri* at the EUC/EIC interface is much lower than the SEC/EUC interface, and therefore the shear instabilities and turbulent mixing are even stronger there. In addition to the mean zonal jets, the equatorial waves with period between 20–100 days also make a considerable contribution to the sub-inertial *S*^2^ (with a ratio of ~1/3). Our further analysis shows that the 20–100 day velocity shear has a good correspondence with the local zonal wind stress with the correlation coefficient of −0.44 near the surface (Supplementary Fig. 2b). Additionally, lead-lag correlations between them indicate very prominent downward-propagating signals from near surface down to ~300 m depth (Supplementary Fig. 2b). Considering that the 20–100 day *S*^2^ is strongest near the surface and monotonically decreases with depth (Fig. 3a, c), the above results suggest that the equatorial waves are most possibly originated from the intra-seasonal wind forcing near the equator. Although *S*^2^ of the equatorial waves is smaller than the mean zonal jets, they can help trigger instabilities through lowering *Ri* and thus enhance turbulent mixing in these marginally stable currents, especially at the strongly sheared SEC/EUC and EUC/EIC interfaces. Given that these two interfaces are located above and below the main thermocline, respectively (Fig. 4), their combined mixings would accelerate blending of the upper-thermocline and lower-thermocline waters, potentially modulating the upper-ocean heat budget of the Pacific warm pool and influencing the evolutions of monsoon and ENSO.

In contrast to the equatorial peak, both the off-equatorial (12–14°N) and subtropical *S*^2^ peaks (20–22°N) are primarily associated with NIWs. The enhanced near-inertial *S*^2^ at these two peaks are, however, generated by different mechanisms. For the off-equatorial peak, it is demonstrated to be caused by PSI of the diurnal ITs, which generates high-mode NIWs with both up- and down-going energy even in the absence of wind forcing. Although PSI of the semi-diurnal ITs near 28–29°N has been widely reported^9,42,53–56^, direct evidence of the PSI for diurnal ITs is rare due to the lack of specifically designed observations^25,57,58^. As a result of the elevated finescale turbulent shear here, the parameterized *K*_ρ_ showed an increase of 120–300% (incorporate the results of two methods) than the surrounding latitudes, which is comparable to the previously estimated *K*_ρ_ enhancement due to PSI by the semi-diurnal ITs^59^. Compared with the energetic 28–29°N latitudes of Pacific, however, the 12–14°N latitudes has much lower EKE and less near-inertial WW input^31,40^. In such quiescent off-equatorial region, the PSI-generated shear-containing NIWs, therefore, may provide an important route for mixing and vertical exchange of heat and materials.

With respect to the subtropical peak, the increased near-inertial *S*^2^ may to a substantial degree be associated with AE’s inertial chimney effect due to the strong regional eddy activities. Based on the ADCP data from the eddy-resolving mooring array, our study confirms the existence of critical layer for NIWs trapped in the baroclinic AEs. Such a critical layer was first theoretically predicted by Kunze^43^, but has been scarcely observed before^10^. Our observations also suggest that although AEs are favorable for the down-ward propagation of wind-driven NIWs, they may contribute little to the subtropical deep-ocean mixing, because most of high-mode waves break and dissipate near the critical layers at ~200 m depth and can seldom reach the deep ocean (Fig. 7f; Supplementary Fig. 4b). In addition to AE’s inertial chimney effect, other mechanisms such as nonlinear eddy-NIW interaction and spontaneous NIW generation were also suggested to play important roles in enhancing the near-inertial energy and hence turbulent mixing in the eddy-rich regions^10,60–63^. In the scenario of nonlinear interaction mechanism, the energy exchange between eddies and NIWs is highly associated with the strain of eddy fields^60,64^. Because no significant correlations were found between the strain rate of eddies (from altimeter data) and the near-inertial KE/*S*^2^ here, we do not expect an important role played by this mechanism in the subtropical *S*^2^ peak. For the NIWs induced by the spontaneous generation mechanism, they are different from the wind-forced NIWs in that they have comparable energy in the CW and CCW components^10^. By reexamining Fig. 6c, we indeed find that the ratio of CCW near-inertial *S*^2^ (KE) is slightly higher between 20–22°N than the weak-eddy regions (excluding the 12–14°N PSI latitudes). Therefore, the possibility of spontaneous NIW generation by eddies should exist. However, to what extent can it explain the subtropical *S*^2^ peak cannot be explicitly evaluated at present and should be further investigated in the future. Due to the strong turbulent shear associated with eddies, the parameterized *K*_ρ_ at the subtropical *S*^2^ peak is increased by a factor of 2–8 than the background value. Given that the eddy activities have strong interannual-to-decadal modulations in the STCC region^65,66^, the eddy-mediated turbulent mixing may also have a corresponding low-frequency variability, which can potentially impact the overlying atmosphere through modulating the sea surface temperature.

Although our observations are confined to 143°E of the North Pacific, we expect that the observed latitudinal dependence of finescale turbulent shear and diapycnal mixing as summarized in Fig. 9 are qualitatively valid for the South Pacific and other oceans, since the three latitude-dependent dynamical regimes are a common feature in the world’s tropical-extratropical oceans. Recent model studies have suggested that the latitudinal structure of *K*_ρ_ has an important influence on the simulated climate^4,5^. Given this, our present study highlights the need to take into account the observed latitude-dependent generation processes of turbulent mixing in ocean models’ parameterization schemes to improve their accuracy of climate simulations and predictions.

## Methods

### Moored data

In order to directly observe the full-depth current velocity along 143°E, the 17 NPEIM moorings were equipped with one or two 75 kHz RDI ADCPs at ~500 m depth and several discrete Aanderaa RCMs below that. The ADCPs measured the upper-ocean velocity (above 1000 m or 500 m) every 16 m, while the RCMs provided point velocity measurements in the deep layer. All the ADCPs and RCMs sampled the velocity every half an hour and had continuously worked for at least 13 months until their recovery; as such, they fully resolved mesoscale eddy and internal wave signals (i.e., NIWs and ITs). In addition, temperature chains (consisting of several SBE CTDs and dozens of temperature loggers) and discrete CTDs were also mounted on each mooring to measure the temperature and salinity in the upper and deep oceans, respectively. The temperature chains have a depth-dependent vertical resolution between 10–100 m and have a uniform temporal sampling interval of 5 minutes. Unfortunately, the upper segment of temperature chain (above ~500 m) at the moorings P6-7, P10-12, P14, and P16 was lost due to unknown reasons. More detailed information of the moorings can be found in the Supplementary Table 1. For data processing, all the high-frequency raw data were first hourly averaged. Given that the instrument depth fluctuated with time due to the swing of mooring, to keep the depth consistency for different moorings at different time, all the hourly ADCP and temperature chain data were then linearly interpolated to fixed 10 m vertical bins between near surface (40–60 m) and the ~1000 m (or ~500 m) depth. The processed finescale velocity and temperature data were used to calculate the velocity shear and *Ri* and to estimate the *K*_ρ_ in the present study.

### Finescale turbulent shears

The processed ADCP data were used to calculate finescale turbulent shear (i.e., vertical shear of horizontal velocity) at each mooring site. The finescale turbulent shear squared is defined as $S^2 = |\partial u/\partial z|^2 + |\partial v/\partial z|^2$, where *u* and *v* are the zonal and meridional velocity, respectively. To obtain the frequency-dependent components of *S*^*2*^, we first decomposed the velocity into different frequency bands using the third-order Butterworth filter. Specifically, the velocities within the semi-diurnal, diurnal, and near-inertial bands were band-pass filtered with cutoff periods at 10–14 h, 20–27 h, and $2\pi {\mathrm{/}}(1.18 - 0.80)f_0^{ - 1}$, respectively, where *f*_*0*_ is the local inertial frequency depending on the mooring latitude. Because *f*_*0*_ at P1 (near equator) is close to zero, the near-inertial velocity was not computed at this site. For the sub-inertial velocity, it was obtained through low-pass filtering with cutoff periods at $2\pi /0.5f_0^{ - 1}$ for sites P3–17, but 18 days for sites P1 and P2 (equals to $2\pi {\mathrm{/}}0.8f_0^{ - 1}$at P2). The cutoff period of $2\pi {\mathrm{/}}0.8f_0^{ - 1}$ rather than $2\pi {\mathrm{/}}0.5f_0^{ - 1}$ chosen at P2 is to avoid eliminating the equatorial waves with periods between 18–28 days. We should note that, the exact sub-inertial velocity does not exist at P1 because of the near-zero *f*_0_. This term remains here to represent the low-frequency current velocity (intra-seasonal and mean current). Following the frequency-dependent velocity decompositions described above, different components of *S*^2^ were correspondingly obtained.

### Rotary decomposition

To study vertical propagations of NIWs, the near-inertial velocity was decomposed into components that rotate CW and CCW with increasing depth. This was accomplished by taking vertical Fourier transform of the complex near-inertial velocity profile *u*(*z*)+*iv*(*z*) at each time point and then inversely transforming the positive and negative quadrants, respectively^42^. According to the dispersion relation of linear internal waves, the CW (CCW) component with an upward-propagating (downward-propagating) phase has a downward (upward) energy propagation with time^67^. After being decomposed, the CW-component and CCW-component KE (*u*^2^+*v*^2^)/2) and *S*^2^ for NIWs were respectively calculated.

### Richardson number

The Richardson number (*Ri*), which is defined as *Ri* = *N*^2^/*S*^2^, was calculated using the moored data in the upper ~500 m. Here, *N*^2^ is the squared buoyancy frequency and was computed using the Matlab subroutine of SeaWater Library (http://www.cmar.csiro.au/datacentre/) based on the moored temperature data. Because salinity measurements on the moorings were too coarse, the monthly salinity profiles from the IPRC Argo product (http://apdrc.soest.hawaii.edu/) were used in the *N*^2^ computation. Comparisons between the mooring-based *N*^2^ with that computed from all nearby synchronous Argo profiles only show slight difference, suggesting that the monthly salinity did not cause large bias in the *N*^2^ computation. To ensure that *S*^2^ has the same resolution with *N*^2^ in the *Ri* calculation, it was smoothed over a 20-m bin above ~300 m and over a 40-m bin below that according to the different thermometer spacing on the mooring (Supplementary Table 1). We acknowledge that our calculated *Ri* may have been overestimated as the 20–40 m resolution is generally coarser than the scale on which shear instability and turbulence truly occur. However, this does not impact the main conclusions of our study because the *Ri*-related results are primarily qualitative.

### Wind work

The mixed-layer near-inertial work input by wind can be computed from

1 $$\mathrm{WW} = \vec \tau \cdot \vec u,$$

where $\vec \tau$ and $\vec u$ are the surface wind stress and near-inertial velocity, respectively. Because we had no direct wind observations, the 6-hourly ECMWF (European Center for Medium-Range Weather Forecasts; http://apps.ecmwf.int/datasets/) interim wind stress data were used here. Given that the ADCP velocity measurements in the upper 50 m was mostly absent, we used the mixed-layer slab model to solve the near-inertial velocity in Eq. (1) following Alford et al.^39^. The governing equations of the model are:

2 $$\frac{{\partial u}}{{\partial t}} - fv = \frac{{\tau _x}}{{\rho _0H}} - ru,$$

3 $$\frac{{\partial v}}{{\partial t}} + fu = \frac{{\tau _y}}{{\rho _0H}} - rv,$$

where *H* is the mixed-layer depth, *τ*_*x*_ and *τ*_*y*_ are the zonal and meridional wind stresses, *ρ*_0_ is the seawater density, and *r* is the damping coefficient. The ECMWF 6-hourly wind stress was used to force the model. In the computations, the IPRC monthly Argo mixed-layer depth was used and an empirical coefficient *r *= 0.15*f* was chosen^39^.

### Mesoscale eddies

To investigate the impact of mesoscale eddies on NIWs in the subtropical regime, we first identified all the AE and CE events that influenced the mooring sites based on AVISO altimeter data (http://www.aviso.oceanobs.com/). At each subtropical mooring site, the AE-impacted (CE-impacted) periods were identified with the criterion that *ζ* <−0.05*f* (*ζ*  > 0.05*f*), where $\zeta = \partial v/\partial x - \partial u/\partial y$ is the surface relative vorticity calculated using the AVISO geostrophic velocity. For *ζ* below the sea surface, since the moorings are only available in the meridional direction, we calculated *ζ* using the moored ADCP velocity with an approximation of −0.85 ∂*u*/∂*y*. This relation was established based on the least-square fit between ∂*v*/∂*x *‒ ∂*u*/∂*y* and ‒∂*u*/∂*y* at surface, which are highly related with a correlation coefficient of 0.91. With this relation, the mean *ζ* profile within AEs (CEs) can be obtained by averaging all the −0.85 ∂*u*/∂*y* profiles during AE-impacted (CE-impacted) periods of the moorings (see Supplementary Fig. 3). Note that it is the altimeter-based full *ζ* that was used to identify the mesoscale eddies and make the AE/CE composite for near-inertial KE and *S*^2^ (Fig. 7). The approximated subsurface *ζ* was only used to describe the eddies’ vertical structure and to explain the existence of critical layer for the trapped NIWs.

### Estimation of diapycnal diffusivity

In order to estimate the diapycnal diffusivity (*K*_ρ_) based on the moored data, two finescale parameterization methods were employed here. The first one is the widely-used Gregg-Henyey-Polzin (GHP) parameterization based on the internal wave-wave interaction theory^7,14^. According to this method, when the finescale velocity shear data are available, *K*_ρ_ can be estimated through

4 $$K_{\mathrm{\rho}} = K_0\frac{{\left\langle {V_{{z}}^2} \right\rangle ^2}}{{\,{}_{\mathrm{GM}}\left\langle {V_{{z}}^2} \right\rangle ^2}}h_1\left( {R_\omega } \right)j\left( {\frac{f}{N}} \right),$$

where *K*_0_ = 0.5 × 10^−5^ m^2^ s^−1^ is the referenced diffusivity, $\left\langle {V_{{z}}^2} \right\rangle$ and _GM_$\left\langle {V_{{z}}^2} \right\rangle$ are the shear variance from the observed velocity shear spectrum and the Garrett-Munk (GM) model spectrum, respectively^68^. In the above formula, the terms $j\left( {\frac{f}{N}} \right)$ and $h_1(R_\omega )$ are defined as $j\left( {\frac{f}{N}} \right) = \frac{{f\arccos h(N/f)}}{{f_{30}\arccos h(N_0/f_{30})}}$and $h_1\left(R_{\omega} \right) = \frac{{3\left(R_{\omega} + 1\right)}}{{2\sqrt 2 R_{\omega} \cdot \sqrt {R_{\omega} - 1} }}$, respectively, where *N*_0_ = 5.2 × 10^−3^rad s^−1^, *f*_30_ is the inertial frequency at 30°N, and *R*_*ω*_ is the shear/strain variance ratio. Following the previous studies^14,69^, *R*_*ω*_ is set conservatively to 7 here. To quantify the shear variance $\left\langle {V_z^2} \right\rangle$, we first calculated the shear spectrum using the 320 m-segment hourly velocity data between 90–410 m (see examples of shear spectra in Supplementary Fig. 5). Then, it was obtained by integrating the spectrum from the minimum wavenumber $k_{\min } = \frac{{2\pi }}{{160}}{\mathrm{ rad}} \, {\mathrm{ m}}^{{{ - 1}}}$ to the maximum wavenumber $k_{\max } = \frac{{2\pi }}{{32}}{\mathrm{ rad}} \, {\mathrm{ m}}^{{{ - 1}}}$. The GM shear variance _GM_$\left\langle {V_z^2} \right\rangle$ was computed over the same wavenumber band. For the stratification *N* used in the $j\left( {\frac{f}{N}} \right)$ and GM model, it was calculated based on the hourly temperature data at the moorings with recovered upper-layer temperature chains (see Richardson number in the Methods). With respect to the seven moorings in absence of upper-layer temperature chains (Methods; Supplementary Table 1), *N* was instead estimated using the monthly Argo data. Although the instantaneous diffusivities estimated based on the moored and Argo stratifications showed evident differences at the same mooring site, they actually had very close monthly distributions and the differences between their annual mean values were only between 3–12% (Fig. 8a). This good agreement gives us confidence to trust the annual-mean latitudinal pattern of the estimated *K*_ρ_ with the Argo-based stratifications.

Given that the GHP parameterization may be invalid for the equatorial region where turbulent mixing is closely related to the strongly sheared sub-inertial currents rather than the breaking of internal waves, we also adopted a straightforward Richardson number-based parameterization method^50,70^ to independently estimate *K*_ρ_. The formula is in the form of

5 $$K_{\uprho} = K_0 + K_{\mathrm{m}} \cdot \left(1 + Ri/Ri_{\mathrm{c}}\right)^{ - 1},$$

where *Ri*_c_ = 1/4 is the critical *Ri* value for shear instability, *K*_0_ and *K*_m_ are the constant background diffusivity and maximum diffusivity, respectively. By analyzing dozens of microstructure profiles in the low-latitude northwestern Pacific, the recent study of Liu et al.^50^ demonstrated that this finescale *Ri*-based parameterization can well approximate the observed *K*_ρ_ when choosing *K*_0_ = 2.1 × 10^−6^ m^2^ s^−1^ and *K*_m_ = 1.9 × 10^−4^ m^2 ^s^−1^ (determined by nonlinear least-square fit). Here, the same parameters proposed by Liu et al.^50^ were used considering our similar study region. Similar to the results from the GHP method, using the moored or Argo stratifications in Eq. (5) only had very little influence on the estimated annual-mean diffusivities (with differences between 3–14%; Fig. 8b) and therefore the Argo-based *N* was used at the moorings where temperature chains were lost.

## Electronic supplementary material

Supplementary Information

## Data Availability

The altimeter, Argo, and wind data used in this study were obtained from the website of AVISO, APDRC, and ECMWF, respectively. The mooring data and computing codes used in this study are available from the corresponding author upon reasonable request. All figures in this paper were plotted using Matlab.
