# Supplementary material for: Latitude-dependent finescale turbulent shear generations in the Pacific tropical-extratropical upper ocean
Source: Nat Commun. 2018 Oct 5;9:4086. doi: 10.1038/s41467-018-06260-8 (PMC6173738; doi:10.1038/s41467-018-06260-8)
Supplement: Supplementary file 1 — Supplementary Information [file 41467_2018_6260_MOESM1_ESM.pdf]

Supplementary Information

Latitude-dependent finescale turbulent shear generations in the Pacific tropical-extratropical upper ocean

Zhang et al.

Accompanying the article "Latitude-dependent finescale turbulent shear generations in the Pacific tropical-extratropical upper ocean" by Zhang Z.-W., B. Qiu, J.-W. Tian, W. Zhao, and X.-D. Huang

This Supplementary Information includes:

- Supplementary Figures 1-5..Pages 2-6
- Supplementary Table 1..Pages 7-10

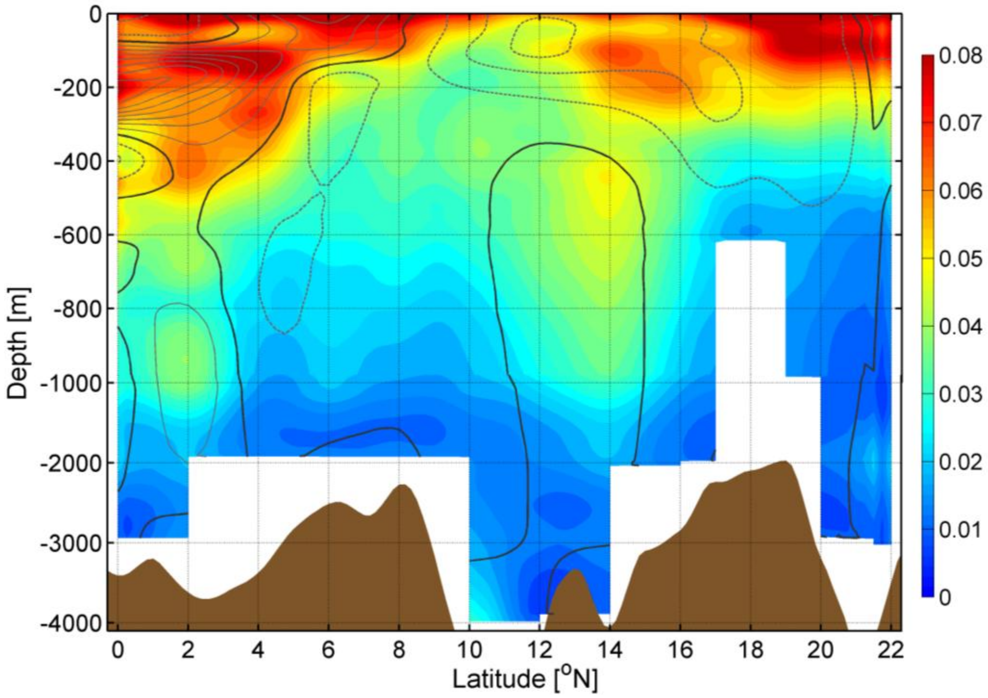

Supplementary Figure 1 | Latitude-depth distribution of mean EKE. The EKE is calculated based on the 20-120 day band-pass filtered velocity and is averaged over the whole observation period. Color shading denotes the root-mean-squared EKE (with unit of m/s). Black and gray contours are the same as those in Fig. 1b of main text that depict the mean circulation.

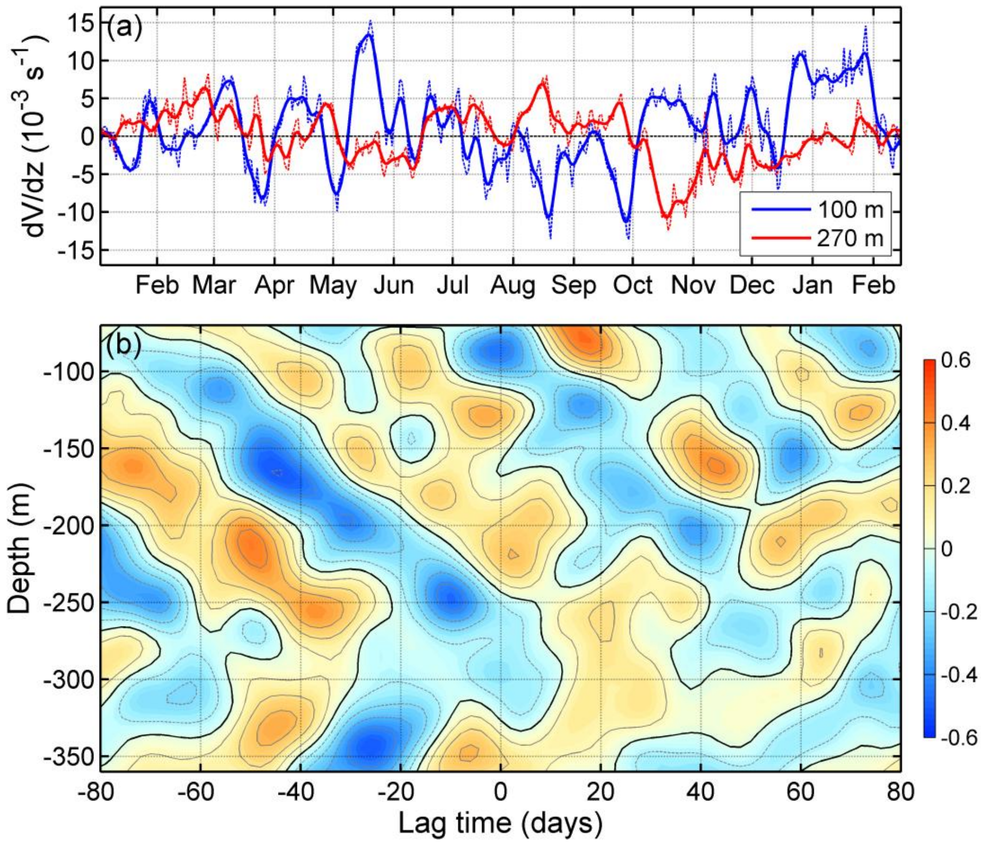

Supplementary Figure 2 | Sub-inertial shear and its correlation with wind. (a) Time series of the meridional sub-inertial shear (V_z) at 100 m (blue) and 270 m (red) depth on the equator**.** Dashed lines denote the original daily-mean series. **(b)** Lag correlations between V_z at different depths and the on-site ECMWF zonal wind stress (see methods). Before calculations, all the time series were firstly 10–120 day band-pass filtered. Colored shadings denote the correlation coefficients (with contour interval of 0.1) and thick black line is the zero contour. Negative and positive time lags indicate that the wind leads and lags the V_z, respectively.

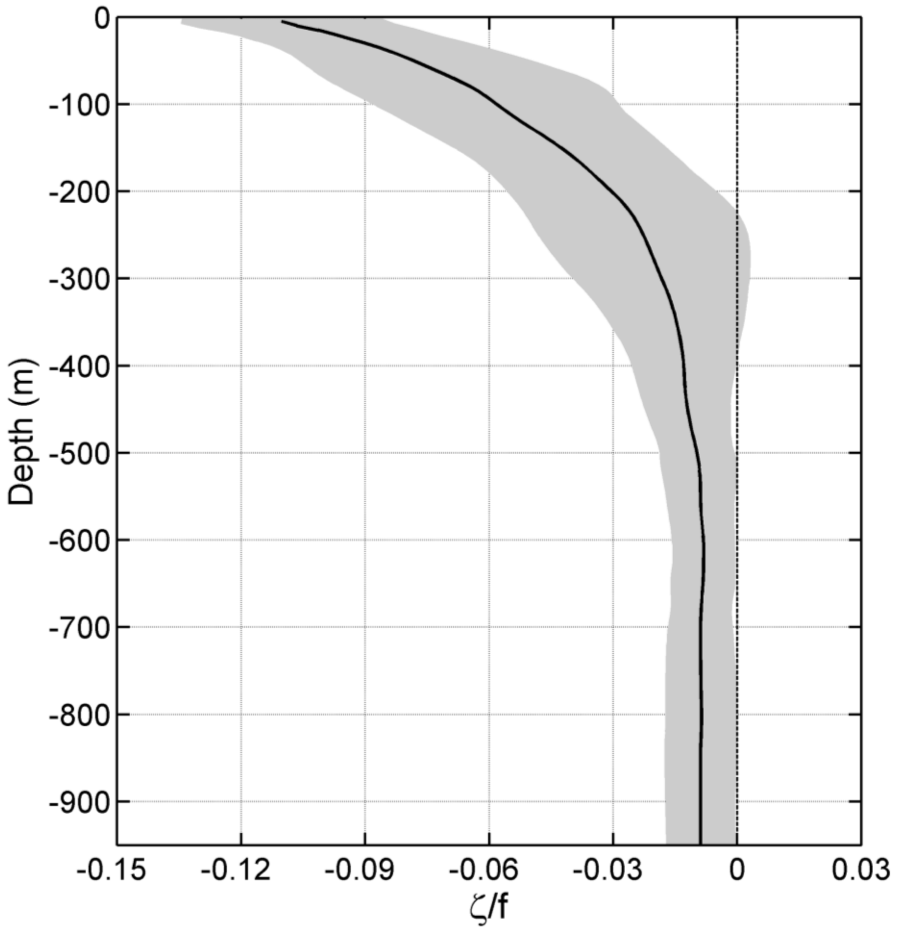

Supplementary Figure 3 | Mean profile of relative vorticity within AEs. Black line is the mean relative vorticity (normalized by f) during the AE periods calculated based on ADCP data from the mesoscale-resolving mooring array (see methods). Gray shading denotes standard deviation of the black line.

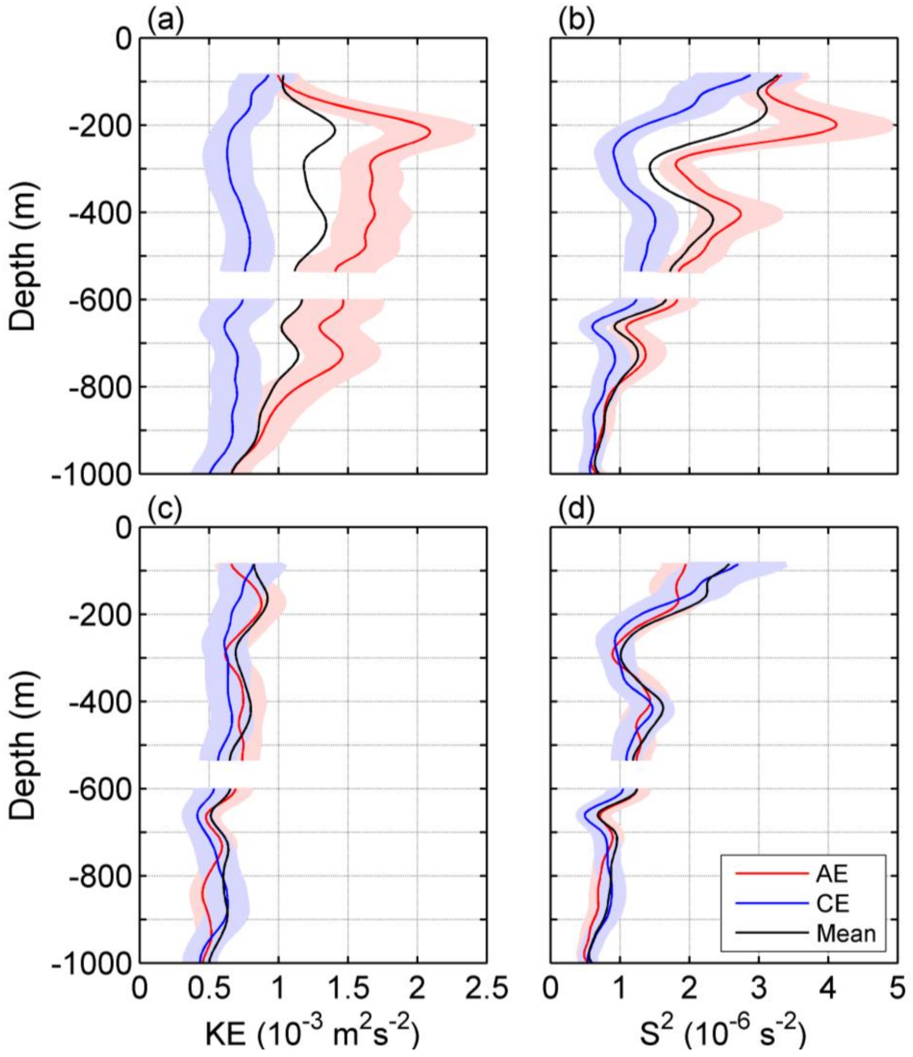

Supplementary Figure 4 | Compositely mean profile of near-inertial KE and S^2 within subtropical eddies. **(a)** Mean near-inertial CW-component KE during the AE- (red line) and CE-impacted (blue line) periods calculated based on the 2.7-year ADCP data at 22°N. Colored shadings denote the corresponding 95% confidence intervals computed using the bootstrap method. Black line denotes the mean CW-component KE during the whole observation period. **(b)** Same as (a) but for the CW-component S^2. **(c, d)** Same as (a, b), respectively, but for the CCW component.

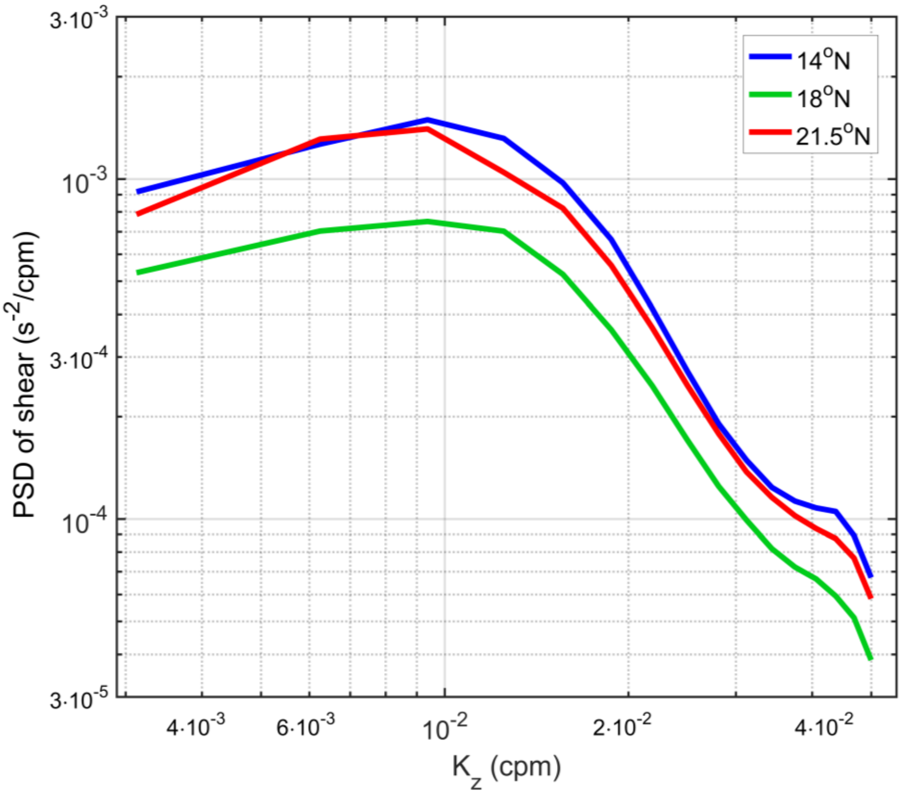

Supplementary Figure 5 | Vertical wavenumber spectra of the velocity. The blue, green and red lines denote the results at 14°N, 18°N and 21.5^{o}N, respectively. The spectrum is the year-long composite result calculated from the velocity in the upper 400 m.

Supplementary Table 1 | Detailed configuration of the NPEIM moorings.

Site Name	Observation Period	Longitude, Latitude	Water Depth (m)	Instrument (looking)[&]	Instrument Depth (m)	Range Depth (m)	Bin Size (m)	Sample Interval (min)	Lost[#] Instruments
P1	Dec/18/2015-<br>Feb/18/2017	142.94 °E,<br>-0.02 °N	3151	CTDs	45,500,2010<br>3020	**	**	5	
				Temperature chains	**	55-295<br>295-495<br>495-995	20<br>40<br>100	5	
				75 kHz ADCP(up)<br>75 kHz ADCP(dw)	520<br>520	50-500<br>540-980	16	30	
				RCMs	2005,3015	**	**	30	
P2	Dec/31/2015-<br>Feb/19/2017	142.99 °E,<br>2.03 °N	3837	CTDs	30,480,1990	**	**	5	
				Temperature chains	**	40-280<br>280-480<br>480-980	20<br>40<br>100	5	
				75 kHz ADCP(up)<br>75 kHz ADCP(dw)	520<br>520	70-490<br>500-1060	16	30	
				RCMs	1985,2990	**	**	30	
P3	Jan/1/2016-<br>Feb/17/2017	143.01 °E,<br>4.02 °N	3566	CTDs	25,480,1990<br>3005	**	**	5	
				Temperature chains	**	35-275<br>275-475<br>485-975	20<br>40<br>100	5	
				75 kHz ADCP(up)<br>75 kHz ADCP(dw)	500<br>500	50-480<br>520-900	16	30	
				RCMs	1985,3000	**	**	30	
P4	Jan/1/2016-<br>Feb/20/2017	143.01 °E,<br>5.97 °N	2587	CTDs	35,536,2008	**	**	5	
				Temperature chains	**	45-285<br>285-485<br>485-985	20<br>40<br>100	5	
				75 kHz ADCP(up)<br>75 kHz ADCP(dw)	490<br>490	40-480<br>510-1040	16	30	
				RCM	2005	**	**	30	
P5	Jan/2/2016-<br>Feb/16/2017	142.99 °E,<br>7.97 °N	2161	CTDs	35,495,2004	**	**	5	
				Temperature chains	**	45-285<br>285-485<br>485-985	20<br>40<br>100	5	
				75 kHz ADCP(up)<br>75 kHz ADCP(dw)	495<br>495	50-480<br>520-1015	16	30	

				RCM	1999	**	**	30	
P6	Jan/3/2016-<br>Feb/21/2017	142.98 °E,<br>9.99 °N	4733	CTDs	35,490,2000<br>4030	**	**	5	CTD and T<br>loggers<br>between<br>35-445m
				Temperature chains	**	45-285<br>285-485<br>485-985	20<br>40<br>100	5	
				75 kHz ADCP(up)<br>75 kHz ADCP(dw)	490<br>490	45-475<br>520-1000	16	30	
				RCMs	1995,3010<br>4025	**	**	30	
P7	Jan/5/2016-<br>Feb/21/2017	142.98 °E,<br>11.99 °N	4139	CTDs	30,480,2000<br>4022	**	**	5	CTD and T<br>loggers<br>between<br>30-440m
				Temperature chains	**	40-280<br>280-480<br>480-980	20<br>40<br>100	5	
				75 kHz ADCP(up)<br>75 kHz ADCP(dw)	480<br>480	50-460<br>510-990	16	30	
				RCMs	1995,3006<br>4017	**	**	30	
P8	Nov/25/2015-<br>Mar/2/2017	143.01 °E,<br>14.08 °N	4027	CTDs	30,490,2010<br>3928	**	**	5	
				Temperature chains	**	40-280<br>280-480<br>480-980	20<br>40<br>100	5	
				75 kHz ADCP(up)	470	40-460	16	30	
				RCMs	1027,2005<br>2964,3923	**	**	30	
P9	Nov/25/2015-<br>Mar/3/2017	143.02 °E,<br>15.88 °N	2965	CTDs	140,595,<br>2010	**	**	5	
				Temperature chains	**	150-390<br>390-590<br>590-1090	20<br>40<br>100	5	
				75 kHz ADCP(up)<br>75 kHz ADCP(dw)	570<br>570	50-560<br>600-1100	16	30	
				RCM	2105	**	**	30	
P10	Nov/26/2015-<br>Mar/3/2017	143.01 °E,<br>16.94 °N	2312	CTDs	80,530,2052	**	**	5	CTD and T<br>loggers<br>between<br>80-490m
				Temperature chains	**	90-330<br>330-530<br>530-1030	20<br>40<br>100	5	
				75 kHz ADCP(up)	510	40-500	16	30	
				RCMs	1047,2047	**	**	30	
P11	Nov/26/2015-<br>Feb/13/2017	142.99 °E,<br>17.94 °N	2183	CTDs	190,645	**	**	5	CTD and T<br>loggers
				Temperature chains	**	200-440	20	5	

						440-640<br>640-1140	40<br>100		between<br>190-600m
				75 kHz ADCP(up)	620	60-610	16	30	
P12	Nov/27/2015-<br>Feb/13/2017	142.87 °E,<br>19.01 °N	2057	CTDs	70,525,1028	**	**	5	CTD and T<br>loggers<br>between<br>70-480m
				Temperature chains	**	80-320<br>320-520<br>520-1020	20<br>40<br>100	5	
				75 kHz ADCP(up)	500	40-490	16	30	
				RCM	1023	**	**	30	
P13	Nov/27/2015-<br>Mar/5/2017	143.09 °E,<br>20.02 °N	3218	CTDs	20,470,2990	**	**	5	
				Temperature chains	**	30-70<br>70-470<br>470-870	20<br>40<br>100	5	
				75 kHz ADCP(up)<br>75 kHz ADCP(dw)	480<br>480	45-465<br>500-990	16	30	
				RCMs	1998,2985	**	**	30	
P14	Nov/27/2015<br>-Mar/6/2017	143.06 °E,<br>20.59 °N	3849	CTDs	40,490,2010<br>3020	**	**	5	CTD and T<br>loggers<br>between<br>40-450m
				Temperature chains	**	50-290<br>290-490<br>490-990	20<br>40<br>100	5	
				75 kHz ADCP(up)<br>75 kHz ADCP(dw)	490<br>490	50-470<br>515-945	16	30	
				RCMs	2005,3015	**	**	30	
P15	Nov/28/2015-<br>Mar/8/2017	142.98 °E,<br>20.96 °N	4038	CTD	25,480,3003	**	**	5	
				Temperature chains	**	35-75<br>75-475<br>475-875	20<br>40<br>100	5	
				75 kHz ADCP(up)<br>75 kHz ADCP(dw)	480<br>480	50-470<br>500-990	16	30	
				RCM	2005,2998	**	**	30	
P16	Nov/28/2015-<br>Mar/7/2017	143.04 °E,<br>21.46 °N	4294	CTDs	40,490,2005<br>4026	**	**	5	CTD and T<br>loggers<br>between<br>40-450m
				Temperature chains	**	50-290<br>290-490<br>490-990	20<br>40<br>100	5	
				75 kHz ADCP(up)<br>75 kHz ADCP(dw)	490<br>490	50-460<br>520-1000	16	30	
				RCMs	2000,3010<br>4021	**	**	30	
P17	Nov/29/2015-<br>Mar/7/2017	143.08 °E,<br>21.89 °N	3495	CTDs	120,574<br>2074,3084	**	**	5	
				Temperature chains	**	130-170	20	5	

						170-570<br>570-870	40<br>100		
				75 kHz ADCP(up)<br>75 kHz ADCP(dw)	565<br>565	50-535<br>600-1050	16	30	
				RCMs	2069,3079	**	**	30	

Note: [&] up and dw denote that the ADCP looks upward and downward, respectively. [#] The uppermost segment of the mooring was cut off due to unknown reasons, resulting in that one CTD and several temperature loggers (i.e., temperature chain) mounted on were lost.
